# Value-added salad dressing enriched with red onion skin anthocyanins entrapped in different biopolymers

**DOI:** 10.1016/j.fochx.2022.100374

**Published:** 2022-06-22

**Authors:** Florina Stoica, Nina Nicoleta Condurache, Iuliana Aprodu, Doina Georgeta Andronoiu, Elena Enachi, Nicoleta Stănciuc, Gabriela Elena Bahrim, Constantin Croitoru, Gabriela Râpeanu

**Affiliations:** a Department of Food Science, Food Engineering, Biotechnology and Aquaculture, Faculty of Food Science and Engineering, Dunărea de Jos University of Galati, 800201 Galați, Romania; b Academy of Agricultural and Forestry Sciences, 61 Marasti Blvd, 011464 Bucharest, Romania

**Keywords:** *Allium cepa* L., Red onion skins, Anthocyanins, Encapsulation, Hydrogels, Salad dressing

## Abstract

In this study, red onion skin extract was used to obtain food ingredients. Complex biopolymeric matrices were dissolved in the anthocyanin-rich aqueous extract, followed by gelation and freeze-drying. Powders were characterized regarding encapsulation efficiency (EE), phytochemical content, color, antioxidant activity, and microstructure. Storage and simulated digestion stability were also assessed. Two powders with high contents of bioactives and antioxidant activity were obtained. The highest EE was acquired for the powder with a higher polysaccharides concentration (V2). In addition, V2 exhibited the best storage stability. The *in vitro* studies demonstrated that increased carbohydrate concentration delivers the best anthocyanins protection. To prove its functionality, V2 was added to a salad dressing. The addition of powder has improved the concentration of biologically active compounds and the antioxidant activity of the salad dressings. These results support the assumption that microencapsulation may deliver bioactives from red onion skin as functional ingredients for value-added foods.

## Highlights

• The microencapsulation of ROS extract was performed by gelation and freeze-drying.
• The powders were rich in valuable antioxidants, with over 80% EE for anthocyanins.
• Good stability of phytochemicals over storage and digestion was noticed.
• V2 powder’s functionality was tested by its addition to salad dressing.
• Our results are promising in developing multifunctional ingredients for foods.

## Graphical abstract

**Figure:**
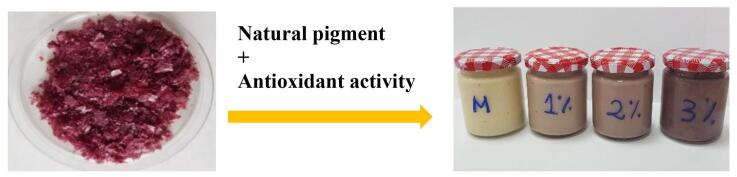

## 1 Introduction

Onion (*Allium cepa* L.) is the main crop, extensively grown worldwide, with a production of around 98 million tons every year. Its consumption has steadily increased since onions have been promoted as a healthy, nutritious, and versatile food (Bedrníček et al., 2020).

Significant quantities of phenolic-rich by-products are being produced in food processing industries. For instance, the European Union produces annually over 500,000 tons of onion by-products, which could be considered an important natural source of polyphenols. Moreover, onions are abundant in phenolic acids, flavonoids, organosulfur compounds, thiosulfates, sugars, and anthocyanins (Ersoy et al., 2019).

The skin or outer layer of red onion contain high content of dietary and phenolic compounds, including flavonoids (quercetin glucosides and anthocyanins) and alk(en)yl cysteine sulfoxides (Celano et al., 2021). Red onion skins (ROS) are renewable raw materials for the extraction of bioactives because they can be used as a source of functional components in value-added products. The use of onion skin may offer the chance to valorize these low-cost by-products from economically relevant perspectives. Likewise, it can have promising applications in food industry and related fields such as cosmeceuticals and biopharmaceutical industries (Gontard et al., 2018).

Anthocyanins are well-known antioxidant compounds. They exert antioxidant capacity by directly scavenging free radicals and indirectly scavenging reactive oxygen species occurring via oxidative stress. Anthocyanins have proved protection against oxidative DNA cleavage, membrane strengthening, and enzyme inhibition. They have many properties that play significant roles in human health. It poses anti-inflammatory, antioxidant, anti-carcinogenic, and anti-obesity properties, inhibiting lipid peroxidation and preventing diabetes (Jiang et al., 2020).

Anthocyanins' sensitivity to temperature, oxygen, light, and pH confines their use, which is why the bioavailability of anthocyanins is very low. Therefore, they need to be protected to bring the desired effects on the target. While encapsulated, the biologically active compounds are entrapped in wall materials to produce microcapsules, restricting their reactivity with environmental factors and improving their bioavailability as well as their shelf life (Sharif et al., 2020). Encapsulation can be used for bioactive compounds protection during storage and the gastrointestinal tract to control the release of the core materials for their absorption and action. Accordingly, the core and shell material are deliberated as the structural items of encapsulation (Tarone et al., 2020). The type of wall material is fundamental, as it determines the encapsulation efficiency (EE) and powder characteristics. Several biopolymers can be used as wall materials, including pectin, carboxymethyl cellulose (CMC), and proteins. Pectin has gelling properties, high biocompatibility, and biodegradability, and is susceptible to solubility and hydrophobicity upgrades (Chen et al., 2015). Proteins are nontoxic, cheap, biocompatible, biodegradable, and are used as matrix materials due to their film-forming and gel-forming capacities and their favorable interactions with polyphenols. Soy protein isolates (SPI) are considered suitable encapsulating materials for different bioactive compounds for their availability and lower cost. Compared to proteins from animal sources are more renewable raw materials (Tang & Li, 2013).

One of the widely applied techniques for the encapsulation of biologically active compounds is freeze-drying. Freeze-drying is an excellent mechanism for drying heat-sensitive compounds that creates high-quality products, permitting the preservation of the functionality of bioactive compounds, with minimal thermal and oxidative degradation (Ozkan et al., 2019). Gelation is another promising encapsulation technique since it is low cost, non-toxic, and uses renewable natural wall materials. Hydrogels are three-dimensional networks formed by randomly linked polymeric chains through physical or chemical interactions (Batista et al., 2019). The hydrogels obtained by physical interactions are based on non-covalent bonds (hydrogen bonds, ionic/electrostatic interactions, van der Waals forces, or hydrophobic interactions) produced through changing pH, heating/cooling, high-pressure processes, or adding ions. The hydrogels obtained by chemical interactions are based on covalent cross-links, which are more stable due to stronger bonds (Khalesi et al., 2020). Natural macromolecules, synthetic polymers, or a combination of both can be used to generate hydrogels. Natural polymers comprising proteins or different polysaccharides, on the other hand, have better biocompatibility, biodegradability, and toxicity than synthetic ones. Hydrogels with controlled, resistant, and nontoxic qualities can be made using copolymers and multi-component blends, such as the protein-carbohydrates complex (Batista et al., 2019). Proteins - polysaccharides interactions play an important role in the gelation mechanism. Proteins have a positive charge at pH values below the isoelectric point (4.5 for SPI), and most polysaccharides have a negative charge. Therefore, stronger protein-polysaccharide complexes are expected, which should be difficult to reverse, thus resulting in stronger gels (Falsafi et al., 2022). Cold-set gels are ideal candidates for designing functional foods that preserve heat-sensitive bioactives. Furthermore, the cold-induced gelation technique allows for more control over the shape, structure, and texture of the produced gel (Abaee et al., 2017).

The study aimed to microencapsulate the anthocyanins from ROS extract using a combination of biopolymeric matrices, through gelation, and freeze-drying techniques. SPI with pectin and CMC were used as coating materials in different concentrations due to their capability to form hydrogels with three-dimensional network structures. The resulting two experimental powder versions were comparatively characterized concerning EE, total anthocyanin content (TAC), total flavonoid content (TFC), total polyphenol content (TPC), and free radical scavenging activity. The bioavailability of anthocyanin in the simulated gastrointestinal conditions, the powders' microstructural pattern, color, and storage stability of the phytochemicals were assessed. In order to test the functionality, the most promising powder, with a higher concentration of polysaccharides (V2) was added to a recipe of a foodstuff (salad dressing), which was further characterized. The results attained in this study could bear an important benefit concerning exploiting the bioactive ability of phytochemicals, considering developing food products with enhanced functional properties.

## 2 Materials and methods

### 2.1 Materials

Soy protein isolate (90 % protein content), was purchased from Kemin Industries (Herentals, Belgium). Ethanol 96 % was purchased from Titolchimica (Italy). Apple pectin, sodium carboxymethyl cellulose, HPLC purity methanol, 2,2-diphenyl1-picrylhydrazyl (DPPH), Folin–Ciocâlteu reagent, 6-hydroxy-2,5,7,8-tetramethylchromane-2-carboxylic acid (Trolox), gallic acid, sodium nitrite solution, glacial acetic acid, potassium chloride solution, sodium acetate solution, sodium hydroxide, aluminum chloride, sodium carbonate, hydrochloric acid, sodium bicarbonate were acquired from Sigma-Aldrich (Steinheim, Germany).

### 2.2 Anthocyanin extraction from ROS

Red onions (*Allium cepa L.*) were bought from a local market (Galați, Romania) in August 2021. The bulbs were peeled using a sharp cutter to remove the red outer dry layers. Then, the skins were washed with distilled water and, next, soaked up on paper towels. Skins were then dried at 40 °C using a conventional oven (Stericell 111, MMM Medcenter, USA) for 2 h, up to a moisture content of 11.0 %. After being pulverized with a domestic blender (mean particle diameter of ∼ 1 mm), ROS were stored in a hermetically closed container at an average temperature of 4 °C.

Ultrasound-assisted extraction (UAE) method was used to extract the anthocyanins from ROS (Albishi et al., 2013). Shortly, 1 g of ROS was suspended in 15 mL of a solvent mixture comprised of 70 % ethanol solution acidified with glacial acetic acid (ratio 6:1, *v/v)*. The extraction was conducted utilizing a sonication bath (MRC. ltd, Holon, Israel), at 25 °C, with a frequency of 40 kHz for 20 min. Further, the extraction was repeated three times in order to get anthocyanins-enriched extracts, while the supernatants were collected and centrifuged for 15 min at 6000 rpm and 4 °C. The resulting supernatants were concentrated to dryness at 40 °C, under reduced pressure (AVC 2–18, Christ, Shropshire, UK), and then used for the characterization and experiments dealing with powder development.

### 2.3 Extract characterization

The obtained extract was characterized regarding TAC, TFC, and TPC contents and antioxidant activity, using the pH differential, aluminum chloride, Folin-Ciocâlteu, and DPPH free radical-scavenging methods, respectively (Stănciuc et al., 2017).

The TACs of ROS extract are expressed in mg cyanidin 3-*O*-glucoside (C3G)/g dry weight (DW). The TFCs are reported as mg Quercetin equivalent (QE)/g DW, the TPCs as mg Gallic acid equivalents (GAE)/g DW, and the antioxidant activity as mM Trolox equivalents (TE)/g DW. All phytochemicals were evaluated using a UV–vis spectrophotometer with data analysis software (Libra S22, Biochrom, UK).

### 2.4 Microencapsulation of anthocyanins from ROS extract

A modified method detailed by Serrano-Cruz et al., (2013) was used to encapsulate the anthocyanins from ROS in hydrogels. Hydrogels, also mentioned as aqua gels, are three-dimensional network structures consisting of polymeric chains linked by chemical bonds (Batista et al., 2019).

In our study, two experimental powders were obtained using SPI with CMC, and pectin wall materials. For powder V1, 4 % SPI, 2 % CMC, and 2 % Pectin, were solubilized in ultrapure water and stirred electromagnetically for 4 h at 40 °C and 350 rpm. Powder V2 was obtained by mixing 2 % SPI with 4 % CMC and 4 % Pectin in ultrapure water and stirred for 4 h at 40 °C and 350 rpm. Next, to ensure full hydration, both mixtures were stored at 4 °C overnight. Then, ROS extract (25 mg/mL) was added to each combination and homogenized until complete hydration for 3 h at 25 °C and 400 rpm. The measured pH of the mixtures was 3.5. Further, the samples were frozen at −70 °C and freeze-dried (CHRIST Alpha 1–4 LD plus, Germany) at −42 °C under a pressure of 10 Pa for 48 h. In the end, the powders were packed into plastic tubes with light protection and stored in the refrigerator at 4 °C for further analysis.

### 2.5 Powder characterization

#### 2.5.1 Encapsulation efficiency

The obtained powders were phytochemically examined regarding the TAC, TFC, TPC, and antioxidant activity against DPPH free radicals. The EE of the powders was determined by assessing the surface anthocyanin content (SAC) and total anthocyanin content (TAC) of the powders (Stănciuc et al., 2017).

Briefly, for TAC analysis, 200 mg of each powder was mixed with 5 mL of a mixture of methanol, acetic acid, and water (25:4:21, v/v/v). The blends were vortexed (1 min) and sonicated to break the microcapsules (DCG-80H, MRC Scientific Instruments ltd., Holon, Israel) for 20 min at 40 ± 1.0 °C. Additionally, the supernatants were centrifuged at 14000 rpm for 10 min at 4 °C.

For SAC, 200 mg of each powder was mixed with 5 mL of ethanol: methanol mixture (1:1, v/v) and vortexed with a BV 1000 Vortex mixer (Benchmark Scientific Inc., Edison, NJ, USA) for 1 min. Finally, the samples were centrifuged for 10 min at 14000 rpm and 4 °C. The TAC and SAC of the resulting supernatants were examined utilizing the pH-differential method (Stănciuc et al., 2017). EE was determined using Eq. (1).

(1) EE (%) = (TAC - SAC)/TAC × 100.

TAC—Total Anthocyanin Content; SAC—Surface Anthocyanin Content.

#### 2.5.2 Colorimetric analysis

The color of the microencapsulated extract was measured using the Chroma Meter CR-410 equipment (Konica Minolta Sensing Americas Inc., Ramsey, NJ, USA). According to the CIELAB system, the L* indicates the lightness (0 = black and 100 = white), while a* and b* are coordinates for green (-a*)/red (+a*), and blue (-b*)/yellow (+b*). The chroma or color intensity (Chroma = $\sqrt{{{(a}^{*})}^{2}+{{(b}^{*})}^{2}}$), as well as the hue angle (Hue angle = 360 + arctan(b*/a*) for quadrant IV (+a*, -b*)), which indicate the color of the powders [0° (pure red color), 90° (pure yellow color), 180°(pure green color) and 270° (pure blue color)], were also calculated (Dag et al., 2017).

#### 2.5.3 Storage stability

The powders were filled in Falcon plastic tubes, and kept in the dark at 4 °C. The bioactive contents (TAC, TFC, and TPC) and antioxidant activities were measured, as previously described, over 28 days of storage.

#### 2.5.4 Confocal laser scanning microscopy (CLSM)

The confocal laser scanning microscopy analysis was used to determine the structural characteristics of the microcapsules containing bioactive compounds from ROS extract. The images were captured with a Zeiss confocal laser scanning system (LSM710) equipped with several types of lasers, such as diode laser (405 nm), Ar-laser (458, 488, 514 nm), DPSS laser (diode-pumped solid-state – 561 nm), and HeNe-laser (633 nm). The powders containing bioactive compounds from ROS extract were observed with a 20 × apochromatic objective, zoom 1, while the obtained 3D images were rendered and processed by ZEN 2012 SP1 software (Black Edition).

#### 2.5.5 *In vitro* digestibility

*In vitro* digestion was performed as described earlier by Minekus et al., (2014) by utilizing the static model that simulates gastric and intestinal digestion. The gastric digestion was simulated using gastric juice (SGJ) with porcine pepsin (40 mg/mL in 0.1 M HCl, pH = 3.0). The simulated intestinal digestion was performed using intestinal fluid (SIF) containing pancreatin from the porcine pancreas (2 mg/mL in 0.9 M sodium bicarbonate, pH = 7). During the entire experiment, the samples were incubated in a shaker (Medline Scientific, Chalgrove, Oxon, UK), at 150 rpm and 37 °C, for 2 h/digestion. Every 30 min, a 0.2 mL mixture for each sample was gathered for TAC estimation to finally assess the percentage of anthocyanins released out of the experimental powders.

### 2.6 Preparation and characterization of a value-added salad dressing

The V2 powder was added as an ingredient in a salad dressing at three different ratios (1 %, 2 %, and 3 %), such as to exploit the multifunctional properties of the obtained powder. The recipe involved mixing of olive oil (12 mL), apple cider vinegar (12 mL), natural tahini sesame paste (40 mL), pepper (0.1 mg), salt (0.8 mg), V2 powder (1–3 %), and water (40 mL). The control sample was salad dressing without powder addition (C). Products were homogenized to be uniform in terms of color and texture, then allowed to stand at 25 °C for 1 h to equilibrate, and afterward stored in the refrigerator. The samples were coded as D1, D2, and D3 based on increasing powder concentration (1 %, 2 %, and 3 %), respectively. The value-added salad dressings were examined for phytochemicals’ stability and radical scavenging activity over 14 days of storage at 4 °C.

#### 2.6.1 Texture analysis

The texture analysis was achieved using a Brookfield Ametek CT3 Texture Analyzer, applying the Texture Profile Analysis (TPA) method. The samples were packed into cylindrical plastic containers of 43 mm in diameter and 30 mm high. A cylindrical acrylic probe (25.4 mm diameter, 25 mm high) was used to penetrate the samples to 15 mm depth. Two penetration cycles, at 1 mm/s speed and 0.067 N load cell were applied. The force–deformation parameters were processed with TexturePro CT V1.5 software in order to determine the textural parameters: firmness, adhesiveness, cohesiveness, and springiness. Three replicates for each sample were made.

#### 2.6.2 Rheological analysis

Rheological measurements were performed using a control-stress rheometer (AR2000ex, TA Instruments, ltd, New Castle, DE) which allows the temperature control by means of integrated heating and cooling Peltier jacket.

The rheological properties of the dressing samples were evaluated under oscillatory flow in small amplitude conditions (strain sweep and frequency sweep tests) and forced flow conditions (stepped flow tests). The strain sweep tests were performed at a constant frequency of 1 Hz over an oscillatory strain range of 0.01 %–100 %, the frequency sweep tests at an increasing frequency from 0.1 to 100 Hz, and at strain values in the linear viscoelastic region (LVR) and the stepped flow tests at a shear rate increasing from 0.1 to 100 1/s. All measurements were carried out at 20 °C by using a plate geometry with a diameter of 40 mm and a gap of 1000 mm. When testing the dynamic viscoelastic properties the storage modulus (G′) and loss modulus (G″) were recorded, whereas in the case of the steady shear viscosity tests, the shear stress and apparent viscosity were registered.

### 2.7 Statistical analysis

The data stated in this present study include the average of triplicate analyses and were reported as mean with standard deviation. After checking the normality and homoscedasticity tests (Minitab 19, Minitab Inc., PA, USA), the data were analyzed utilizing a one-way analysis of variance (ANOVA). Tukey test was used to identify significant differences between the results (*p < 0.05*).

## 3 Results and discussion

### 3.1 ROS extract characterization

In the present study, the extraction process was carried out using the UAE method, with ethanol 70 % and glacial acetic acid as a solvent (in a 6:1 ratio, v/v). The ROS extract was characterized in terms of biologically active compounds content and antioxidant activity. The extract displayed a TAC of 1.37 ± 0.02 mg C3G/g DW, with a TFC of 194.38 ± 1.57 mg QE/g DW and a TPC of 133.51 ± 1.01 mg GAE/g DW. The radical scavenging activity of the ROS extract showed a value of 43.86 ± 0.47 mM TE/g DW. Albishi et al. (2013) reported lower phytochemical values. They found a TAC of 10.04 ± 0.91 mg C3G/100 g DW, a TPC of 25.45 ± 0.74 mg GAE/g DW, a TFC of 20.22 ± 0.39 mg QE/g DW, and the antioxidant activity of 0.152 ± 0.004 mM TE/g DW after the UAE extraction of bioactives from ROS using methanol–acetone–water (7:7:6, *v/v/v*). Katsampa et al. (2015) found higher TAC of 1.87 ± 0.39 mg C3G /g DW and a lower TPC of 61.47 ± 14.19 mg GAE/g DW in the ROS extracts obtained with the UAE method using 90 % (*w/v*) aqueous glycerol, as solvent. All these authors reported variable results for the phytochemical content of ROS extract due to the variability of raw material and extraction conditions.

A preliminary High Performance Liquid Chromatography (HPLC) analysis, also presented in our previous study (Stoica et al., 2021), showed that the predominant anthocyanins found in ROS extract were cyanidin 3-*O*-laminaribioside and cyanidin 3-*O*-(600-malonyl-laminaribioside). Besides these two, cyanidin 3-*O*-glucoside; cyanidin 3-*O*-(3′′-malonylglucoside), peonidin 3-*O*-glucoside, cyanidin 3-*O*-(6′′-malonylglucoside), peonidin 3-*O*-malonylglucoside and cyanidin 3-*O*-dimalonylaminaribioside were also found, but in lower concentrations, <8 %. Our results are in agreement with other studies reported by Celano et al. (2021).

### 3.2 Powders’ phytochemical characterization and encapsulation efficiency

Encapsulation efficiency, a significant quality parameter, is the percentage of the bioactive that is successfully entrapped and held into the shell material of the microcapsule (Tarone et al., 2020).

According to Table 2, the EE of the samples was significantly influenced by the encapsulating wall materials used since the mixture prepared with a high concentration of polysaccharides resulted in higher anthocyanin EE (*p < 0.05*). Thus, the EE significantly increased with the carbohydrate polymers concentration, ranging from 85.92 ± 1.14 % for V1 to 97.36 ± 2.61 % for V2 (*p < 0.05*). This increase could be explained by the polysaccharides' disruptive effect on the protein/polyphenol complex in a matrix during encapsulation (Thongkaew et al., 2014). Bourvellec and Renard (2012) explained this phenomenon by the ability of polysaccharides to encapsulate phenolic compounds, competing with the binding to proteins. They also stated that ionic polysaccharides like pectin are known as effective inhibitors of polyphenol/protein complexation.

Our reported data are in agreement with other studies. Elsebaie & Essa (2018) reported a high EE of 94.30 ± 1.50 % of ROS extract in SPI and maltodextrin. Additionally, Tao et al. (2017) combined various shell materials (whey proteins, gum Arabic, maltodextrin, and β-cyclodextrin) for the encapsulation of blueberry extracts and reported anthocyanins’ EE with values exceeding 82 %.

In our study, the resulting powders obtained by gelation technique followed by freeze-drying, displayed different phytochemical profiles (Table 2). Significantly higher TAC, TPC, TFC, and antioxidant activity values were acquired for V2 than V1 (*p < 0.05*). It can therefore be stated that higher concentrations of polysaccharides than proteins as wall materials allowed the retention of higher concentrations of biologically active compounds from ROS extract. Stănciuc et al. (2017) published interesting results with similar behavior. The authors encapsulated anthocyanins from grape skins in whey proteins isolate and two different polysaccharides by freeze-drying. They achieved higher concentrations of TPC and antioxidant activities for the powder with higher polysaccharides concentration. Notwithstanding, both powders are characterized by a high antioxidant activity with high entrapment efficiencies. Thus, the selected wall material combinations successfully encapsulated the phytochemicals from ROS extract. Moreover, from the data presented in Table 2, it can be concluded that when considering the effect of freeze-drying on the phytochemical profile of the powders, several factors should be examined, such as the core-to-coating ratio, interaction effects as well as chemical properties.

### 3.3 Colorimetric analysis

The L*, a*, b*, hue angle, and chroma color parameters measured for the two experimental powders at the initial moment and after 28 days of storage are shown in Table 1. The highest L*(lightness) value was obtained for powder V2. After 28 days of storage, lightness decreased significantly (*p < 0.05*) for both powders. Regarding the parameter a* that describes the tendency approaching red, V2 had the highest a* value due to the high anthocyanins content. Parameter a* and chroma increased significantly during the storage time. The increase of a* parameter during storage may be correlated with anthocyanidins released from proanthocyanidins and forming of anthocyanin-derived pigments that stabilized the red-colored flavylium (Moser et al., 2017).

**Table 1:** Color parameters (L*, a*, b*, Hue angle, and Chroma) of the microencapsulated powders.

Sample	Storage time (days)	L*	a*	b*	Hue angle	Chroma
V1	0	35.40 ± 0.02^aB^	9.76 ± 0.03^bB^	−1.33 ± 0.02^bA^	359.86 ± 0.03^bA^	9.85 ± 0.03^bB^
	28	26.07 ± 0.17^bB^	18.46 ± 0.45^aB^	−0.52 ± 0.16^aA^	359.97 ± 0.01^aA^	18.47 ± 0.47^aB^
V2	0	35.55 ± 0.07^aA^	10.17 ± 0.04^bA^	−1.68 ± 0.01^bB^	359.83 ± 0.02^bB^	10.30 ± 0.03^bA^
	28	26.91 ± 0.17^bA^	18.85 ± 0.21^aA^	−1.13 ± 0.06^aB^	359.94 ± 0.02^aB^	18.88 ± 0.21^aA^

**Table 2:** The storage stability test of the powders.

Sample	Storage Time (days)	TAC (mg C3G/g DW)	TFC (mg QE/g DW)	TPC (mg GAE/g DW)	Antioxidant Activity (mM/g DW)
V1	0	1.42 ± 0.04^bB^	129.49 ± 0.74^aB^	116.15 ± 1.12^aB^	50.78 ± 0.06^aB^
	28	1.60 ± 0.03^aB^	124.30 ± 0.50^bB^	104.06 ± 0.21^bB^	49.30 ± 0.28^bB^
V2	0	1.83 ± 0.05^bA^	133.50 ± 1.47^bA^	123.96 ± 1.08^bA^	53.21 ± 0.32^bA^
	28	2.06 ± 0.02^aA^	138.07 ± 1.02^aA^	128.84 ± 0.27^aA^	58.32 ± 0.13^aA^

Negative b* parameter values are an indicator for the blue shades in powders. The highest b* values were obtained for V1, indicating the carrier color contribution to powder b* values. Regardless of storage time, the negative values of the b* parameter in all samples are applied to lightly alkaline conditions when utilizing soy proteins. The b* values increased significantly (*p < 0.05*) over time for all samples. The results were consistent with those reported previously by Tsali & Goula, (2018), who associated increased b* values with the loss of copigmentation effects, accompanied by the formation of pyranoanthocyanins. The results obtained for a* and b* parameters display that all data were located in the fourth quadrant (+a*, -b*), indicating a tendency to red and blue, characteristic of anthocyanins found in the powders.

The chroma parameter increased significantly (*p < 0.05*) for both powders during storage, V1 having the highest degree of color saturation, which is a desirable characteristic. The hue angle values for all samples are closer to 360°, displaying that the main color was red. The hue angle recorded a slight increase for all tested samples during storage.

For each color parameter and powder, values that do not share the same superscript lowercase letter are significantly different concerning time at p < 0.05. Samples that for each color parameter and storage time do not share the same superscript uppercase letter are significantly different at p < 0.05.

### 3.4 Storage stability test of the powders

The storage stability results of powders, concerning polyphenol, flavonoid, and anthocyanin contents as well as the antioxidant activity was presented in Table 2**.**

For each tested phytochemical and each storage time (0, 28 days), values that do not share the same lowercase letters are statistically different at p < 0.05 based on the Tukey method. For each tested phytochemical and each tested powder, values that do not share the same uppercase letters are statistically different at p < 0.05 based on the Tukey method.

From Table 2, it can be observed that V2 showed higher phytochemical stability, leading to an increase in TPC by approximately 4 %, whereas, in V1, a decrease in TPC by 10 % was found. The TAC of both powders displayed an increase of approximately 11 % throughout the 28 days of storage (*p < 0.05*). The TFC increased by almost 3 % for V2, while they decreased by nearly 4 % for V1. Concerning the antioxidant activity, V1 exhibited a 3 % decrease after 28 days of storage, while V2 presented a 9 % increase in the antioxidant activity. Our findings are in good agreement with other studies. Moser et al., (2017), who encapsulated the biologically active compounds from the red grape juice with protein and maltodextrin blends, declared good stability of the anthocyanins at low temperatures (5 °C) for 150 days. Moreover, Mansour et al., (2020) revealed that the combination of SPI and gum Arabic with 0.05 % anthocyanin extract from red raspberry had the highest retention percent of 60.26 % during 60 days of storage at 25 °C.

From Table 2, a slight phytochemical content fluctuation over time can be noticed. However, we could conclude that the used combination with a higher concentration of CMC and pectin successfully encapsulated the anthocyanins from ROS extract, providing them good stability.

### 3.5 Morphological structure of the powders

**Fig. 1:**
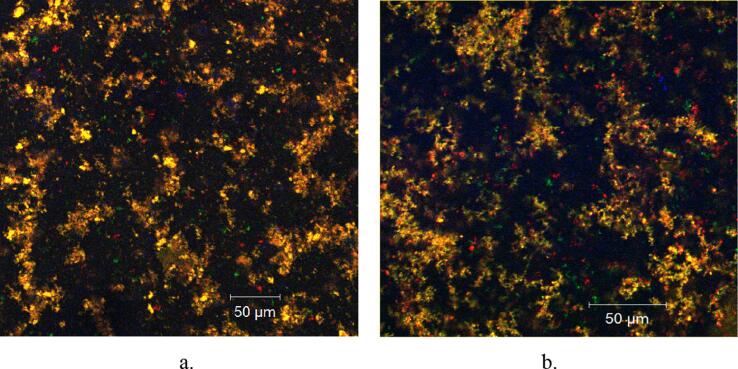
Structure of the powders V1 (a) and V2 (b) by CLSM analysis.

The CLSM analysis displayed relevant characteristics regarding the morphology and structural pattern of the powders. The images were obtained through point-by-point scanning, at a high resolution within a broad field, with the help of digital focusing. The two fine powders are similar from the morphological point of view. Both are in fine microparticles, with a diameter of 1–2 µm, which shows a high aggregation capacity into compact clusters. As many studies states, anthocyanins are extremely sensitive *ex vivo* to many factors: pH, temperature, light, oxygen, metal ions, and intra- or intermolecular association with other compounds (Woodward et al., 2009). Due to that fact, the emission range was exceedingly variable but predominantly between 450 and 570 nm. At pH = 3.5, as the powders were prepared, the anthocyanin peaks were at 520–560 nm (in the green-yellow domain, as seen in Fig. 1) (Liu et al., 2018).

### 3.6 *In vitro* digestion of anthocyanins

**Fig. 2:**
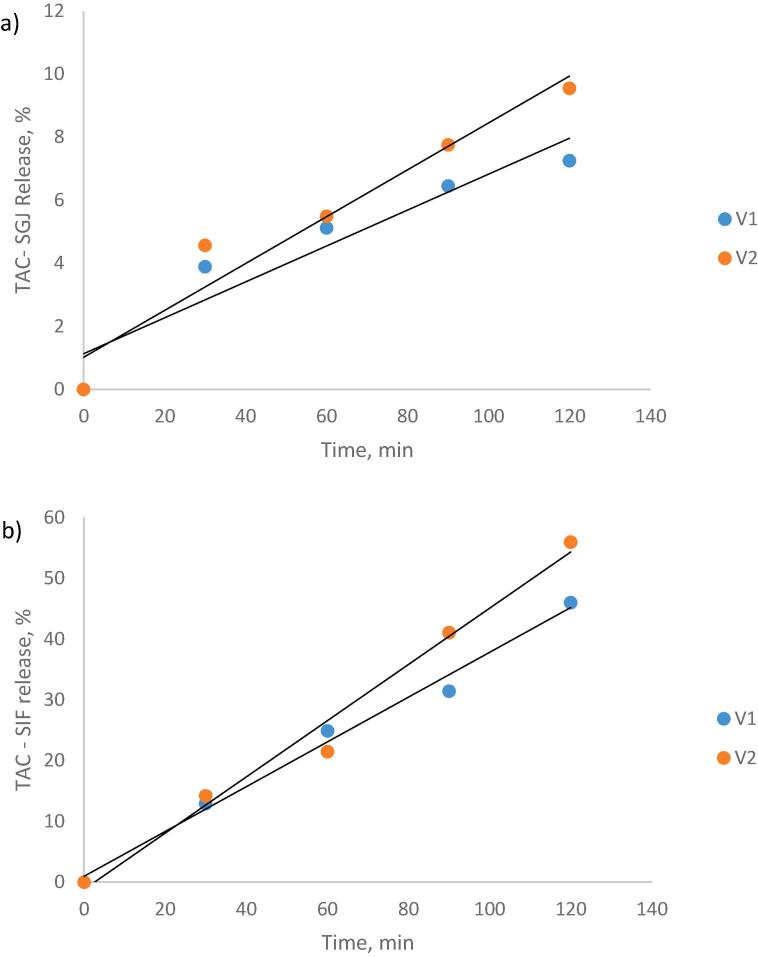
Anthocyanin release from the microcapsule powders during the simulated *in vitro* gastric (a) and intestinal (b) digestion.

The gastro-intestinal behavior of anthocyanins under simulated digestion for 4 h is shown in Fig. 2. The results acquired for *in vitro* digestibility under SGJ and SIF displayed that the picked coating materials exerted a protective effect on the anthocyanin. Thus, a protective effect of biopolymeric matrices on anthocyanins was observed in the gastric phase, while a release was observed in the intestinal phase.

The powders exhibited a slight increase of the anthocyanin content during gastric digestion in a time-dependent manner, with approximatively 7 % for V1 and 9.5 % for V2 after 120 min. This fact suggests that almost 90 % of the anthocyanins remained in the microcapsules. In SIF, after 120 min of digestion, the anthocyanins' release reached a maximum of 46 % for V1 and 56 % for V2. This fact suggests that a significant amount of anthocyanins remained in the microcapsules, being able to reach the colon and being absorbed by the body.

These findings comply with other studies. Wu et al., (2020) encapsulated the anthocyanins from blueberry extract with gelatin, SPI, maltodextrin, and Arabic gum. The authors revealed that soy proteins encapsulation delayed the release of anthocyanins and significantly increased colonic accessibility. In addition, SPI and gum Arabic used for the encapsulation of raspberry extract conferred good release under simulated gastric and intestinal digestion compared to unencapsulated anthocyanin extract (Mansour et al., 2020).

Therefore, it can be appreciated that the microparticles coated by CMC, P, and SPI are resistant to the gastro-intestinal environment. Thus, a higher anthocyanin protective effect can be observed by the matrix having a higher concentration of polysaccharides (V2).

### 3.7 Characterization of value-added salad dressing

**Fig. 3:**
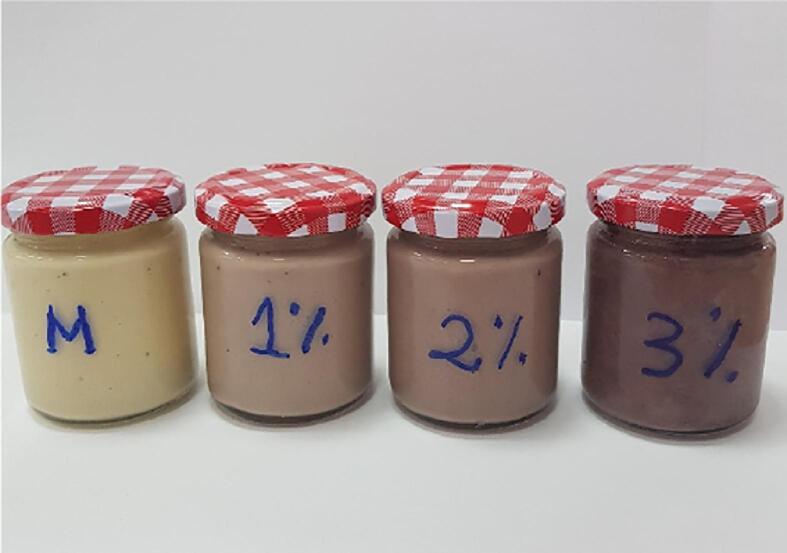
Value-added salad dressing samples with different percentages of encapsulated ROS extract: M (control), 1% (D1), 2% (D2), and 3% (D3).

To test V2 powder as a food ingredient with multiple functionalities, different percentages of 1 % (D1), 2 % (D2), and 3 % (D3) were added to a salad dressing (Fig. 3). The obtained food products were analyzed regarding the bioactive's stability (TAC, TFC, TPC, and antioxidant activity) during storage at 4 °C for 14 days. The phytochemical content of the experimental products is shown in Table 3.

**Table 3:** Phytochemical profile of added-value salad dressing and stability during 14 days of storage.

Sample	Time, days	TAC, µgC3G/g DW	TFC, mg QE/g DW	TPC, mg GAE/DW	Antioxidant activity, mM TE/g DW
Control	0	–	1.59 ± 0.04^a^	0.99 ± 0.03^a^	2.61 ± 0.19^a^
	14	–	1.61 ± 0.02^a^	1.01 ± 0.02^a^	2.98 ± 0.05^b^
D1 (1 %)	0	12.59 ± 0.16^a^	1.88 ± 0.01^b^	1.56 ± 0.01^b^	7.20 ± 0.08^c^
	14	12.33 ± 0.96^a^	2.23 ± 0.01^c^	1.62 ± 0.01^c^	7.68 ± 0.11^d^
D2 (2 %)	0	22.46 ± 0.20^b^	2.62 ± 0.02^d^	2.01 ± 0.02^d^	10.52 ± 0.12^e^
	14	21.24 ± 0.51^c^	2.71 ± 0.03^e^	2.11 ± 0.01^e^	10.72 ± 0.02^f^
D3 (3 %)	0	32.92 ± 0.11^d^	3.14 ± 0.02^f^	2.62 ± 0.03^f^	12.37 ± 0.28 ^g^
	14	31.30 ± 1.07^e^	3.51 ± 0.03 ^g^	2.71 ± 0.01 ^g^	12.90 ± 0.05 ^h^

Values with different letters in the same column are significantly different (p < 0.05).

As shown in Table 3, adding the microcapsules into the salad dressing considerably increased the TAC, TFC, TPC, and antioxidant activity compared to the control sample. As expected, increasing the powder concentration resulted in a significant increase in the phytochemical contents (p < 0.05). As a result, the salad dressing samples had TACs ranging from 12.59 ± 0.16 µg C3G/g DW to 32.92 ± 0.11 µg C3G/g DW. A higher TFC of the salad dressings was acquired compared to control, with 15 % in D1, 39 % in D2, and 49 % in D3, respectively. Further, when compared to the control, the TPC in the formulated salad dressings was 37 % in D1, 51 % in D2, and 62 % in D3 higher. The antioxidant activity of the enriched salad dressings was positively influenced by the bioactive components extracted from ROS, presenting higher values than the control samples. The data in Table 3 confirm the added value of the developed salad dressings with encapsulated ROS extract by increasing the phytochemical content and antioxidant activity.

After 14 days of storage, no significant decrease (p > 0.05) was found in the anthocyanin contents of D1, in contrast with D2 and D3, where a significant decrease of approximately 5 % was observed (*p < 0.05*). Furthermore, a significant increase during the storage period was found in the flavonoid and polyphenol contents of all three salad dressings. From Table 4, a significant increase (p > 0.05) in the antioxidant activity of all tested salad dressings over the storage time can be noticed. It is probably due to the release of some other compounds apart from anthocyanins, such as phenolics and flavonoids from microcapsules. Therefore, an increase of 6 %, 2 %, and 4 % in antioxidant activity was found for D1, D2, and D3, respectively. Our results comply with Ivanova et al., (2018), who also reported bioactive contents' decrease over the storage time from dairy dessert enriched with encapsulated cornelian cherry, chokeberry, and blackberry juices.

**Table 4:** Texture and rheological properties of the salad dressings prepared with different amounts of microcapsule powder (0% - Control, 1% - D1, 2% - D2, and 3% - D3).

		Sample			
		Control	D1	D2	D3
***Texture properties***					
Firmness, N		0.32 ± 0.04^b^	0.40 ± 0.03^b^	0.41 ± 0.02^b^	0.84 ± 0.06^a^
Adhesiveness, mJ		0.87 ± 0.12^b^	1.17 ± 0.30^b^	1.19 ± 0.12^b^	3.57 ± 0.13^a^
Cohesiveness		0.70 ± 0.06^a^	0.71 ± 0.01^a^	0.72 ± 0.01^a^	0.8 ± 0.01^a^
Springiness, mm		7.73 ± 0.65^a^	8.11 ± 0.01^a^	8.17 ± 0.08^a^	8.49 ± 0.20^a^
***Rheological properties***					
Critical strain, %		2.03 ± 0.002^d^	2.54 ± 0.008^c^	3.19 ± 0.016^b^	5.17 ± 0.087^a^
Viscosity (Pa·s) at shear rate of	1 1/s	4.44 ± 0.23^dA^	8.70 ± 0.02^cA^	10.28 ± 0.09^bA^	27.93 ± 1.90^aA^
	10 1/s	1.18 ± 0.05^dB^	1.97 ± 0.03^cB^	2.63 ± 0.01^bB^	8.21 ± 0.004^aB^
	100 1/s	0.288 ± 0.012^dC^	0.59 ± 0.003^cC^	0.96 ± 0.02^bC^	2.66 ± 0.013^aC^

### 3.8 Texture analysis of value-added salad dressing

Table 4 shows the values of the textural parameters for salad dressing samples. Hardness is expressed as the maximum force registered for the first penetration cycle (Bourne, 2002). As it can be noticed in Table 4, the addition of encapsulated ROS resulted in firmness increasing. This might be due to the increased concentration of dry matter and proteins, which leads to a denser structure of dressing. At low addition levels (1 % and 2 %) the firmness did not show significant differences, while the 3 % addition induced a more accentuated firmness increase. This is associated with a lower spreadability of the samples. Manakla et al., (2019) reported similar firmness values for the black sesame spreads with xanthan gum and Tanislav et al., (2021) for sunflower tahini.

Adhesiveness is defined as the work required to overcome the attractive forces between the product and the testing probe (Bourne, 2002). The lowest adhesiveness value of 0.87 mJ, was registered for the control sample, while for the other samples higher values were found. Manakla et al., (2019) explained the increased values of black sesame paste adhesiveness as a result of high viscosity of the aqueous phase. In our samples, the composition of encapsulated ROS might determine these results.

Cohesiveness is a measure of how well the product withstands a second deformation relative to its resistance under the first deformation (Bourne, 2002). Higher cohesiveness values were registered for the samples with encapsulated ROS addition. This could be correlated with the increased values of proteins content, which induce a denser structure of the samples.

Springiness is related to the amount of deformation that is recovered after the first penetration cycle. It is associated with a solid behavior of the samples. For the analyzed salad dressing samples, springiness showed higher values with higher powder concentration. These results are correlated with the other textural parameters values.

The texture analysis revealed that the addition of encapsulated ROS enhanced the textural properties of the salad dressing, proportionally with the concentration.

Within a row, means values with different superscript lowercase letters are significantly different (p < 0.05).

Within a column, means values with different superscript uppercase letters are significantly different (p < 0.05).

### 3.9 Rheological behaviour of the value-added salad dressing

The influence of microcapsule powder addition on the rheological properties of the dressing samples was assessed by measuring the dynamic viscoelastic and flow behavior. The strain sweep tests were first employed to determine the LVR where the internal structure of the dressing samples is not influenced by the amplitude of the applied strain (Tunick, 2011). The addition of increasing amounts of V2 powder to the salad dressing resulted in the increase of the critical strain value (γ_c_), beyond which the samples lose the ability to exhibit linear viscoelastic behavior (Table 4).

**Fig. 4:**
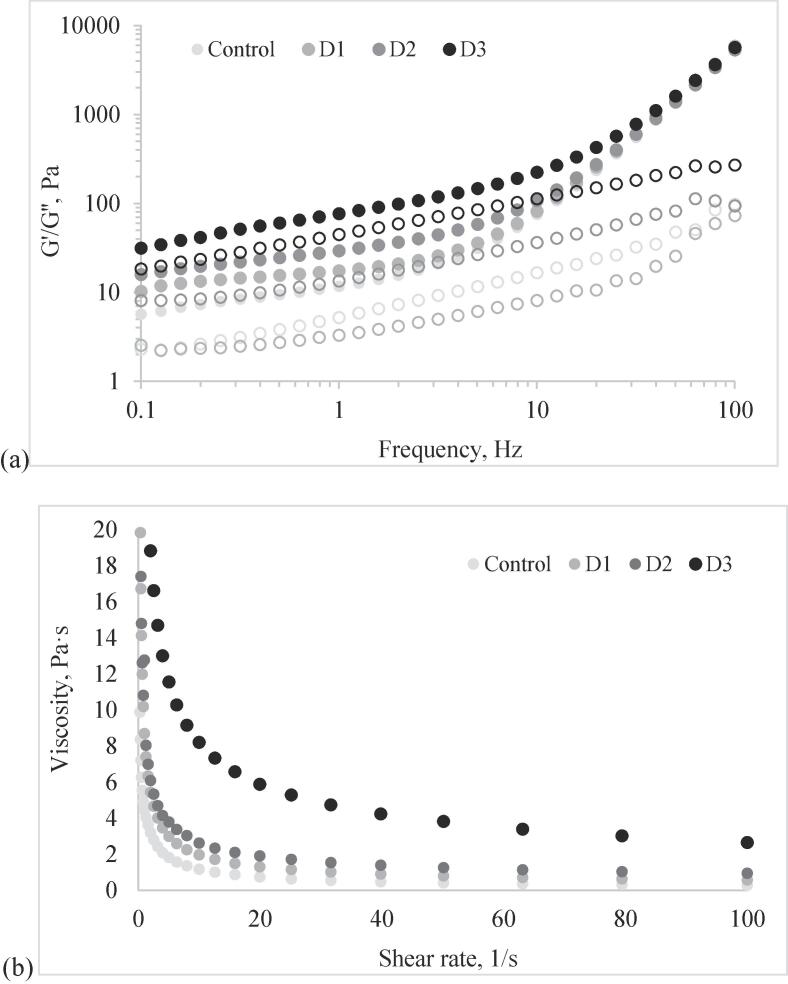
Rheological behavior of the dressing samples with different amounts of microcapsule powder (0% - Control, 1% - D1, 2% - D2 and 3% - D3) under (a) frequency sweep (G′- storage modulus - represented with full symbols and G″- loss modulus represented with empty symbols) and (b) forced flow conditions.

The results of the frequency sweep tests indicated the absence of the strong steric repulsive forces in all investigated samples. Regardless of the microcapsule addition level, the samples exhibited solid-like behavior, with sub-unitary tan (delta) and G’ dominating over the G’’ values at all frequencies (Fig. 4). Diftis et al., (2005) suggested that this trend is specific to the complex networks including droplets, which are unable to rearrange in such manner to accommodate the strain within the period of one oscillation. The addition of the V2 powder most probably allowed new types of intermolecular interactions among the constituents of the complex matrix, such as the interaction between polysaccharides and proteins or lipids. Anyway, considering the high frequency dependence of the complex viscosity observed in case of all investigated samples, one could assume that the addition of microcapsules powder to the dressing did not alter the overall interaction forces ensuring the aggregation of the droplets within the network (Diftis et al., 2005).

The viscosity of the samples was further measured while running steady flow sweep tests. Regardless of the microcapsule powder addition, the apparent viscosity decreased while gradually increasing the shear rate from 0.1 to 100 1/s, suggesting that all samples exhibited shear-thinning behavior. The reduction of the viscosities caused by the gradual breakdown of the semi-solid structure and alignment of the macromolecules of the system with the direction of shearing is more pronounced in the first part of the tested shear rate domain (up to 10 1/s) and riches a plateau when shearing at higher rates (Fig. 4). All samples are in semi-solid condition, with droplets which are prone to deformation and disruption leading to network breaking at shear rate increase, and therefore to the reduction of the viscosity (Chang et al., 2017). Regardless of the shear rate, the V2 powder addition to the dressing determined the significant increase (p < 0.05) of the viscosity (**Table 5**), suggesting the synergistic efficiency of the powder in limiting the flow behavior of the complex matrix.

## 4 Conclusions

This study’s results revealed that the microencapsulated ROS extract has a high content in biologically active compounds that hold a high antioxidant activity. The ROS extract was successfully encapsulated into biopolymeric matrices based on soy proteins, carboxymethyl cellulose, and pectin with an entrapping efficiency of 85.92 ± 1.14 % for V1 and 97.36 ± 2.61 % for V2, respectively. The resulting powders showed high phytochemical contents and free radical scavenging activity. The storage stability test during 28 days at 4 °C revealed high stability of anthocyanins, preserving the antioxidant activity for the powder with the highest concentration of polysaccharides (V2). The confocal laser scanning microscopy analysis revealed that the two powders present similar morphological characteristics, both appearing as fine microparticles with a high aggregation capacity into compact clusters. The color parameters established that red was the predominant color of the powders; though, the increase of b* parameter value in the storage period demonstrated the decrease of co-pigmentation effects. In addition, the selected matrices provided a protective effect during the simulated digestion. Accordingly, the mixture of polysaccharides-proteins presents a high potential for protecting anthocyanins. Our study indicates that the addition of various concentrations of ROS powder to salad dressing formulation allows an improvement of various nutritional properties compared with the control sample. The results obtained in this study could offer the opportunity to use the encapsulated anthocyanins from ROS as functional ingredients for developing new recipes that promote health. Therefore, from the functional and technological point of view, the suggested powder bears added value in foods in terms of color, texture, and antioxidant activity.

## Declaration of Competing Interest

The authors declare that they have no known competing financial interests or personal relationships that could have appeared to influence the work reported in this paper
